# Chlorophyll *a* fluorescence, under half of the adaptive growth-irradiance, for high-throughput sensing of leaf-water deficit in *Arabidopsis thaliana* accessions

**DOI:** 10.1186/s13007-016-0145-3

**Published:** 2016-11-14

**Authors:** Kumud B. Mishra, Anamika Mishra, Kateřina Novotná, Barbora Rapantová, Petra Hodaňová, Otmar Urban, Karel Klem

**Affiliations:** Global Change Research Institute, The Czech Academy of Sciences, v. v. i, Bělidla 986/4a, 603 00 Brno, Czech Republic

**Keywords:** Chlorophyll fluorescence transients, Drought, Whole plant rosettes, Natural accessions, Non-invasive methods, Plant phenotyping

## Abstract

**Background:**

Non-invasive and high-throughput monitoring of drought in plants from its initiation to visible symptoms is essential to quest drought tolerant varieties. Among the existing methods, chlorophyll *a* fluorescence (ChlF) imaging has the potential to probe systematic changes in photosynthetic reactions; however, prerequisite of dark-adaptation limits its use for high-throughput screening.

**Results:**

To improve the throughput monitoring of plants, we have exploited their light-adaptive strategy, and investigated possibilities of measuring ChlF transients under low ambient irradiance. We found that the ChlF transients and associated parameters of two contrasting *Arabidopsis thaliana* accessions, Rsch and Co, give almost similar information, when measured either after ~20 min dark-adaptation or in the presence of *half of the adaptive growth*-*irradiance.* The fluorescence parameters, *effective quantum yield of PSII photochemistry* (Φ_PSII_) and *fluorescence decrease ratio* (*R*_FD_) resulting from this approach enabled us to differentiate accessions that is often not possible by well-established dark-adapted fluorescence parameter *maximum quantum efficiency of PSII photochemistry* (*F*_V_/*F*_M_). Further, we screened ChlF transients in rosettes of well-watered and drought-stressed six *A. thaliana* accessions, under *half of the adaptive growth*-*irradiance*, without any prior dark-adaptation. Relative water content (RWC) in leaves was also assayed and compared to the ChlF parameters. As expected, the RWC was significantly different in drought-stressed from that in well-watered plants in all the six investigated accessions on day-10 of induced drought; the maximum reduction in the RWC was obtained for Rsch (16%), whereas the minimum reduction was for Co (~7%). Drought induced changes were reflected in several features of ChlF transients; combinatorial images obtained from pattern recognition algorithms, trained on pixels of image sequence, improved the contrast among drought-stressed accessions, and the derived images were well-correlated with their RWC.

**Conclusions:**

We demonstrate here that ChlF transients and associated parameters measured even in the presence of low ambient irradiance preserved its features comparable to that of measured after dark-adaptation and discriminated the accessions having differential geographical origin; further, in combination with combinatorial image analysis tools, these data may be readily employed for early sensing and mapping effects of drought on plant’s physiology via easy and fully non-invasive means.

## Background

Sustainable agriculture for feeding the growing human population is a major global challenge [1]. Global warming and consequential erratic climate extremes can further decrease crop yields, and, thus, it may be extremely difficult to fulfill the needed food supplies [2, 3]. Among the many biotic and abiotic stresses responsible for yield losses, drought predominates over others, and, thus, it is a major focus of research in the field [4–8]. Drought is initiated by reduced natural precipitation that activates osmotic stress in plants, causing short term responses reducing water loss, and long term responses modifying metabolic, biochemical, physiological, morphological, and developmental processes including decreases in shoot and increases in root growth [9, 10]. Different species develop different avoidance and tolerance strategies to survive drought; in the former case, plants preserve high water status by enhancing water absorption and/or reducing transpiration, whereas in the latter case, plants maintain turgor pressure and continue metabolism even at low water potential by protoplasmic tolerance or synthesis of osmoprotectants, osmolytes or compatible solutes [10–13]. Therefore, the severity of drought is species-specific and depends, among others, on the developmental stage of the plants.

From a technical and scientific perspective, identification, quantification, and monitoring of drought are extremely complex and difficult; however, they are highly desirable for the screening of tolerant and high yielding genotypes for breeding programs. The general drought tolerance assay, based on survival of plants, is statistically simple but its accuracy is questionable [14]. The quantitative methods, such as relative leaf water content (RWC) or leaf water potential are, however, laborious and time consuming [15]. *Plant phenotyping network* has now initiated several programs for exploitation of numerous non-invasive image-based sensors that may help in rapid characterization of plant traits by decoding genetic information, necessary for sustainable agriculture [16, 17]. These technologies monitor plant growth and biophysical processes rather than their survival; they include visual imaging to gauze the dynamic aspects of morphology, architecture and growth rate [18], thermal imaging to scan stomatal responses [19], hyperspectral imaging to measure pigments and their activities [20], magnetic resonance imaging to study root architecture and physiology [21], and ChlF imaging to study dynamics of photosynthetic performance [22, 23]. Integrated use of these technologies has potential to speed up progress for the better understanding of plant performance by linking gene functions and environmental responses with various biochemical pathways, metabolisms, and processes [24]. Several phenotyping tools and methods are being used with a more practical and a holistic approach; further, automatic phenotypic platforms have vastly improved the screening capacity. Also the focus of research has already been broadened from single plants in controlled environment to real life applications, i.e., many plants in robust greenhouses and under field situations [reviewed in 24–26].

Among the emerging technologies capable of high-throughput screening of diverse plant traits under challenging environmental situations, ChlF transient is highly informative as it responds quickly to changes in both photochemical and non-photochemical processes [27, 28]. ChlF-based methods are highly appropriate for phenotyping since the ideas of redesigning photosynthesis is developing for the active utilization of photosynthetic efficiency to enhance crop yields in the future as the yield potential based on “green revolution” is almost stagnating [29]. Generally, ChlF in vivo is measured after long (~20–30 min) dark-adaptation. This usually allows Q_A_, the primary stable electron acceptor of photosystem (PS) II reaction center, to be fully oxidized, and enables us to measure the *minimal fluorescence* (*F*_O_). On the other hand, the *maximal fluorescence* (*F*_M_) is reached when all Q_A_, and all the electron carriers beyond it, are in the reduced state. The kinetics of the rise from *F*_O_ to *F*_M_, the ChlF transient, is affected by dynamics of the steps involved in PSII and PSI, and beyond, in photosynthesis. In general, when light is absorbed by dark-adapted plant leaves, PSII reaction centers close, and the ChlF yield rises from *F*_O_ to the peak, *F*_P_, during the first seconds of illumination, followed by its decline leading ultimately to a *steady state fluorescence* (*F*_S_) level in a few minutes [30]. The fast rise from O-to-P reveals information about the redox state of electron acceptors of the entire photosynthetic electron transport chain [31–33]. However, the interpretation of slow ChlF transient beyond *F*_P_ is highly complex because several processes, e.g., non-photochemical ChlF quenching, protonation of the thylakoid lumen, ATP synthesis, and activation of the Calvin–Benson cycle, among others, are in action [27, 28, 30]. The technique for measuring ChlF was significantly improved with the addition of saturation pulse methods that helped in resolving photochemical and non-photochemical quenching, and enabled us to measure the photosynthetic performance under field conditions [reviewed in 34]. Further, the availability and affordability of portable fluorometers has revolutionized the photosynthesis research as this method has become widely applicable [28, 35]; it is now being used for non-invasive remote monitoring of different biotic and abiotic stressors having direct or indirect impact on photosynthetic metabolism on plants grown in the laboratory, under controlled environment, and under field conditions [reviewed in 28, 36, 37].

Omasa et al. [38] had introduced an imaging fluorometer for the laboratory use that was further modified by Nedbal et al. [39] to monitor fluorescence in broad sunlight. The images of ChlF parameters have the added advantage to the experimenter for visualizing heterogeneity and spatio-temporal dynamics of physiological processes occurring within large areas [40–42]. The basic ChlF parameters, i.e., single image (*F*_O_, *F*_M_, and *F*_S_), or image derived from arithmetic combination of images [i.e., *F*_V_/*F*_M_, Φ_PSII_ = ($F_{\text{M}}^{\prime }$ − *F*_S_)/$F_{\text{M}}^{\prime }$, and *non*-*photochemical quenching* (NPQ = (*F*_M_ − $F_{\text{M}}^{\prime }$)/$F_{\text{M}}^{\prime }$), where *F*_V_ = *F*_M_ − *F*_O_ and $F_{\text{M}}^{\prime }$ = maximum fluorescence measured under actinic-light], have been evaluated and correlated with changes within leaf physiology, as affected by different stresses [43]. Matouš et al. [44] have incorporated pattern-recognition based advanced statistical approach for the analysis of sequence of time-resolved ChlF images. This approach is based on the performance testing and training of image pixels by using statistical classifiers and feature selection algorithms [45, 46] followed by searching combination of images that can provide high discrimination between groups to be compared [44]. The resulting combinatorial images obtained by this method lack physiological significance; however, they are very powerful and their use was well demonstrated for the early detection of some biotic stresses [44], for species discrimination [47], and for classifying cold tolerance in *Arabidopsis thaliana* accessions [48, 49].

High-throughput measurement is a crucial requirement for the emerging methods to be incorporated in plant phenotyping. ChlF-based methods require prior dark-adaptation of the plant-leaves to be measured for full characterization of the ChlF transients and associated parameters, and this remains one of the main constraint for high-throughput measurements [17, 34, 43]. Because uneven dark-adaptation following light–dark transition may differentially re-oxidize plastoquinone (PQ) pool of thylakoid membranes that influences fluorescence decay [50, 51], and a nested sequential screening (one-by-one measurements after identical dark-adaptation) may take long time for large numbers of plants. Moreover, ChlF transients measured after a long dark adaptation or after a darkening followed by prolonged light exposure (i.e., usually starting before noon) add further risk of being modulated by inactivation of enzymes in Calvin–Benson cycle or downregulation of photosynthetic activity and photoinhibition [52–54]. Simulated high-throughput platforms were used to screen ChlF emission of drought-stress on whole rosettes of *A. thaliana* [22, 55] and on tomato plants [23]. The commonly used dark-adapted ChlF parameter, *F*_V_/*F*_M_, was already demonstrated to be insensitive to detect early drought effects [22, 23]. Other ChlF parameters, e.g., NPQ, Φ_PSII_, and *F*_S_, were shown to be more sensitive as compared to *F*_V_/*F*_M_ as they changed even under mild leaf-water deficit [23, 56, 57]. The parameters *F*_S_ and Φ_PSII_ can be measured in the presence of light without prior dark-adaptation; therefore, they can be adapted for high-throughput screening [56]. However, direct correlation of these parameters with leaf-water deficit is difficult as they are modulated by daily varying environmental stimuli (e.g., light and temperature [58, 59]). In addition, the signals are further influenced by complex processing of absorbed light within the photosynthetic apparatus as well as by drought induced limitations on stomatal conductance, mesophyll conductance to CO_2_ diffusion, leaf photochemistry and biochemistry [56, 60]. Moreover, pattern-recognition based combinatorial imaging has been employed on ChlF transients captured from dark-adapted plants only [44, 45, 47–49]. Time-series images of ChlF transients are spatially heterogeneous and its dynamic features (variations in time-series image pixels) are fully utilized by the algorithms while searching traits of discrimination during training. Thus, the ChlF emission, in principle, has high potential to sense the drought induced systematic changes; however, an effective strategy is required for improving the protocols for measuring ChlF transients and for incorporating efficient post processing methods in order to fully exploit information contained in the image sequences.

In this paper, we have extended the scope of a newly developed phenotyping platform that can automatically screen time-series ChlF images over a 3-m-long transect edge. We have used this system to screen ChlF transients of well-watered and drought-stressed six natural accessions of *A. thaliana*. In order to improve the throughput of this method, we have exploited, for the first time, light-adaptive strategy of plants, and reduced the ambient irradiance to *half of the adaptive growth*-*irradiance* during screening. We propose here a new experimental protocol for the measurement of full ChlF transients without any dark-adaptation and advocate its implication in phenotyping research for screening plant traits in greenhouses and in the diverse and practical environmental situations.

## Methods

### Plant material and its growth conditions

Six natural accessions of *A. thaliana* [Col-0 (Columbia-0) accession, which is genetically related to Gü (Gückingen, Germany); Te (Tenela, Finland); C24 and Co (Coimbra) accessions from Portugal; Nd (Niederzenz, Germany); and Rsch (Rschew, Russia)] were germinated for two weeks and transplanted to cone-type pots (140 mm long; 40 mm diameter) filled with a mixture (1:1, v:v) of substrate and quartz-sand (0–2 mm fraction). Pots with a mixture of substrate and sand were fully watered initially by allowing free capillarity. Seventy plants of each accessions were placed randomly in six trays (each tray had a capacity to grow 98 plants) below the panels of white light-emitting-diode (LED) based light sources (Photon Systems Instruments, Brno, CZ) with an irradiance of ~100 µmol photons m^−2^ s^−1^ (12 h day; 12 h night) on the plant rosettes. Plants were watered every alternate day and supplemented with standard NPK (nitrogen; phosphate; and potassium) fertilizers every two weeks during their growth. The temperature and humidity around the plants were continuously measured every 5 min by temperature/humidity sensors of data loggers (USB-502-LCD, Measurement Computing Corporation, MA, USA). Temperature was controlled by the air conditioning system of the growth room and its average day/night range was ~22.6 ± 1.5/21.0 ± 1.4 °C. The relative air humidity in the vicinity of plants was controlled by a humidifier (LB-4 Steba; Bamberg, DE) and its average day/night range throughout the experiment was 48.3 ± 3.2/52.4 ± 2.5%.

### Strategy for drought induction in *A. thaliana* accessions

Eight week old plants of *A. thaliana* accessions, with fully developed leaf rosettes, were used for the drought experiment. Twenty four plants of each accession were used as well-watered controls. The same number of plants of each accession received drought stress by withholding water for 10 days. Because of different transpiration rates among natural accessions, we monitored soil water content (SWC) of each plant pots by weighing them manually almost every week during 8 weeks of their growth and every third day during the drought experiments. The SWC was kept similar for all accessions with corrected plant weight as mentioned by Granier et al. [61]. The SWC of the used mixture of substrate and sand at retention capacity was ~0.60 g H_2_O g^−1^ dry soil, calculated, before the seedlings were sown, by weighing fully wet and fully dried (4 days at 180 °C) soil. For control plants the SWC was kept at ~80% of their retention limit. The ChlF transients of well-watered and drought-stressed *A. thaliana* accessions were measured and the data were then compared to the RWC of leaves on day 3, 5, 7, and 10 of the induced drought.

### Relative water content (RWC) in leaves of *A. thaliana* accessions

For the objective measurement of leaf-water deficit under drought, RWC in leaves was calculated as (FW − DW)/(TW − DW) × 100; where FW = leaf fresh weight, TW = leaf turgid weight (~24 h in water in darkness), and DW = leaf dry weight (24 h drying at 90 °C). Three leaves from three independent plants of each accession were sampled at midday, and RWC was determined for well-watered and stressed accessions on day 3, 5, 7, and 10 of the induced drought.

### Transect fluorescence imaging platform

**Fig. 1:**
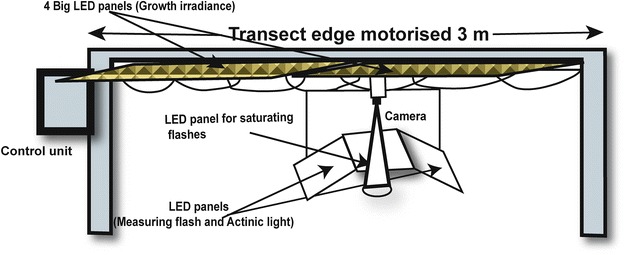
A schematic diagram of transect fluorescence platform. It consists of a motorized 3 m long arm along with a customized open version of imaging fluorometer; it can move with a precision of 1 mm and “capture” chlorophyll *a* fluorescence (ChlF) transients. Open version of fluorometer consists of a CCD camera, two LED panels (~620 nm) that provide both actinic light and measuring flashes, and another LED panel that gives high intensity saturating flashes. The imaging fluorometer as well as movement of camera was fully controlled by protocols through computer. Four white LED panels, each of 1.32 m × 0.32 m, were installed above the transect edge; thus, the plants can even grow right on the platform. For details of fluorometer, see Nedbal et al. [39]

The ChlF transients of the whole plant rosettes were measured by using transect fluorescence imaging platform (Photon Systems Instruments, CZ). A schematic diagram of this platform is shown in Fig. 1. This includes PAM based ChlF imaging system, as described by Nedbal et al. [39]. In this system, imaging CCD camera, along with three LED panels, are movable along the transect edge (~3 m long); it enables us to screen ChlF emission of plants lying below it from the top (~14 mm). Two LED panels (180 × 120 mm each; wavelength 620 nm) were fitted on either side of the transect edge in the vicinity of the camera that could generate both the measuring flash (MF, a very weak intensity of short flash ~30 µs), as well as the actinic light (AL, strong intensity continuous light); while another white LED panel was fitted alongside of the transect edge for generating *saturating flashes* (SF, 0.8 s, ~2000 µmol photons m^−2^ s^−1^). A customized protocol was developed for the movement of camera along the transect axis at well-defined positions with a precision of 1 mm. Further, after capturing time series of ChlF images as programmed upon excitation with combination of lights [MF, SF, and AL generated by respective LED panels], the camera automatically moves to another plant for the next measurement. Four sets of LED based white-light panels (1.32 m × 0.32 m) were installed over the transect edge of the imaging system that provide light with adjustable irradiance between 0 and 1000 µmol photons m^−2^ s^−1^ for growing plants right on the platform.

### Experimental set-up and protocols for screening ChlF emission

To measure ChlF transients without any dark-adaptation, we have tested, for the first time, the possibility to utilize natural light adaptation strategy in plants. Therefore, instead of dark-adapting the plants, we have reduced the ambient irradiance to half of adaptive growth—irradiance (i.e., ~50 µmol photons m^−2^ s^−1^, *low irradiance*) only during the experiment, and screened ChlF transients of all accessions lying below the transect edge, in the presence of *low ambient irradiance*. For screening, we developed a protocol of 248 s, as modified from Mishra et al. [49]; here, the imaging camera moves above the respective plants and stays there for ~50 s, and this is followed by ChlF measurements for another ~198 s before the camera is moved to the next set of plants. The fluorescence measurement protocol uses a weak flash (MF) to measure *steady state fluorescence* in the presence of *low ambient irradiance* [*F*_S_(L_1_), L_1_ ~50 µmol photons m^−2^ s^−1^, for dark-adapted plants *F*_S_(L_1_) = *F*_O_] followed by a saturating flash (SF) to measure light adapted maximum fluorescence ($F_{\text{M}}^{\prime } ,$ for dark adapted plants $F_{\text{M}}^{\prime }$ = *F*_M_). These two parameters were used to calculate the *effective quantum efficiency of PS II* at *low ambient irradiance* [Φ_PSII_(L_1_) = {($F_{\text{M}}^{\prime }$ − *F*_S_(L_1_)/$F_{\text{M}}^{\prime }$}]. After a short-interval of ~27 s, plants were exposed to an *actinic light* (L_2_, ~150 µmol photons m^−2^ s^−1^) for the next ~150 s, and ChlF transients were measured by using a slightly modified standard protocol from that published by Mishra et al. [49]. A saturating flash was used at ~148^th^ s to measure the maximum fluorescence $F_{\text{M}}^{\prime \prime } )$ under actinic light to probe the Φ_PSII_(L_2_) which equals [$F_{\text{M}}^{\prime \prime }$ − *F*_S_(L_2_)]/$F_{\text{M}}^{\prime \prime }$. Further, the fluorescence decrease ratio, *R*_FD_, was calculated as *F*_D_/*F*_S_(L_2_), where, *F*_D_ = *F*_P_ − *F*_S_(L_2_), and *F*_P_ = intensity of fluorescence peak under actinic irradiance. On the day of the screening, plants were acclimated to *half of the growth*-*irradiance* for the first hour of the morning followed by fluorescence measurements for another ~2.48 h for all the 36 plants [six accessions × two groups × three (replicas)] used.

### Combinatorial image analysis for the early diagnosis of drought

Combinatorial image analysis provides an integrated application of classifiers and feature selection methods for the analysis of time series images of ChlF measurements [44, 45, 47–49]. Each ChlF transient data, measured in this experiment consisted of 216 images captured at different time intervals of the experimental protocol, as modified from that of Mishra et al. [49]. In combinatorial imaging, we randomly classify the time series image datasets of the control and the stressed accessions without any bias, and then we calculate the performance of several classifiers, e.g., linear discriminant classifier (LDC), quadratic discriminant classifier (QDC), nearest neighbor classifier (NNC), *k*-nearest neighbors (*k*-NNC), nearest mean classifier (NMC), support vector classifier (SVC), and neural network classifier (NeurC) (for details, see [44, 45, 47]). Further data reduction was performed by implication of sequential forward floating feature selection (SFFS) algorithms [46] and high performing classifiers [for details see 47–49]. The method starts with identifying the fluorescence image out of a total of 216 sets in which the contrast is maximal. This step is followed by finding a second most contrasting image with the same criteria, followed by a homologous search. The process is continued until an optimal classification subset is identified. After identification of three most contrasting images (features), the linear discriminant analysis (LDA) [62] was used to find their most contrasting linear combination. The resulting image was constructed as a linear combination of signals in the given pixel of the three constituent images a.I(t1) + b.I(t2) + c.I(t3). The linear combination expressed a virtual distance of the fluorescence signal of a given pixel from its respective control.

### Tool for the data analysis

Image processing software integrated with the fluorescence imaging system (FluorCam 7, Photon Systems Instruments, CZ) was used to process the captured time-resolved fluorescence images. For statistical analysis of RWC and ChlF parameters, GraphPad Prizm 5 (GraphPad Software-La Jolla, CA, USA) was used. The Matlab software package, version 6.5, with pattern reorganization toolbox (PRTools) was used for combinatorial image analysis.

## Results

### Drought in *A. thaliana* accessions is reflected in RWC

**Fig. 2:**
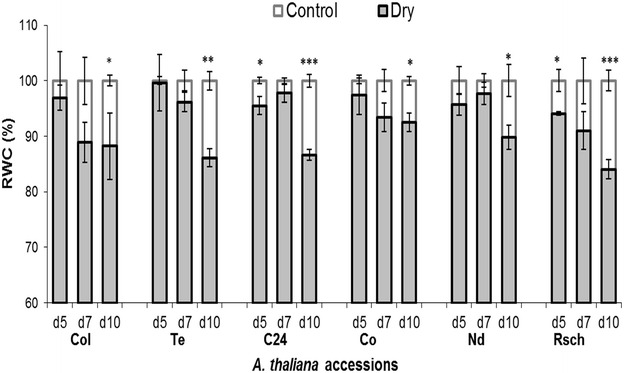
A comparison of relative water content (RWC) in leaves of well-watered (control) versus drought-stressed (dry) *A. thaliana* accessions measured on selected days of drought. Leaves from three different plants were weighted on selected days of drought, and a standard method was used to quantify RWC by measuring fresh weight, turgid weight, and dry weight. The values, given here, are mean from three independent plant leaves ± SE (n = 3). *d5* (day 5), *d7* (day 7), and *d10* (day 10). *Asterisks* denote significant differences between drought-stressed and well-watered plants (*p < 0.05; **p < 0.01; ***p < 0.001; unpaired t test)

A comparison of RWC of leaves from well-watered (control, white background) with those from drought-stressed (dry, gray background) plants is shown in Fig. 2. Until day 3 of the induced drought there was no statistically significant difference in the RWC (data not shown) was obtained; however, on day 5, accessions C24 and Rsch showed a significant decrease (*p* < 0.05) in the RWC of drought-stressed plants as compared to their well-watered counterparts. There were fluctuations in the RWC data of different accessions on day 7 of the induced drought; however, on day 10 of induced drought, analysis of unpaired t test confirmed that RWC of all the accessions was significantly reduced from their respective controls (Fig. 2). The percentage loss of RWC in the investigated accessions on day 10 of induced drought from high to low value was: Rsch (~16%), Te (~14%), C24 (~13%), Col (~12%), Nd (~10%) and Co (~7%).

**Fig. 3:**
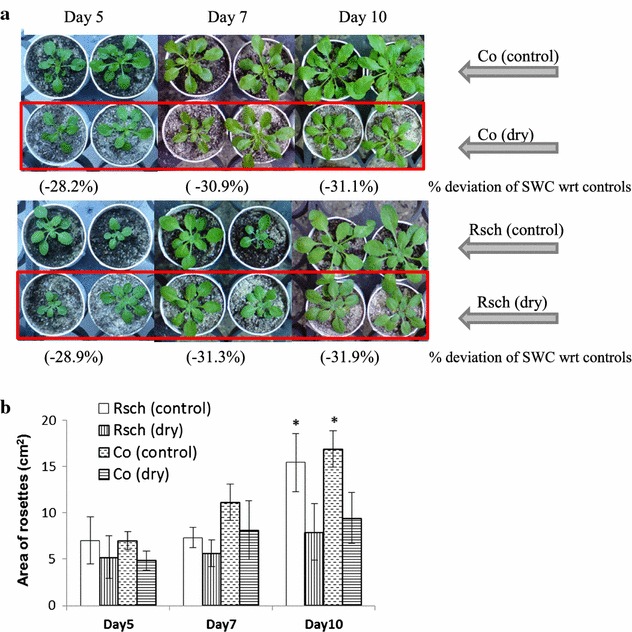
Photographs (**a**) and rosette area (**b**) of well-watered and drought-stressed two contrasting *Arabidopsis thaliana* accessions, Co and Rsch, on day 5, day 7, and day 10 of the induced drought stress. The soil water content (SWC) of the well-watered plants was ~80% of the retention limits of the used substrates and sands, while % deviation of SWC of drought-stressed plants with respect to (wrt) their control counterparts are mentioned below each day of induced drought in the *upper panel*. *Asterisks in the lower panel* denotes significant difference of the rosette area of drought-stressed from well-watered plants (*p < 0.05)

Figure 3 shows photographs (Fig. 3a) and corresponding rosette areas (Fig. 3b) from representative well-watered and drought-stressed plants (on day 5, day 7, and day 10 of the induced drought) of two highly contrasting *A. thaliana* accessions, i.e., Rsch and Co, having maximum (~16%) and minimum (~7%) changes in RWC, respectively. The SWC among the drought stressed accessions was significantly reduced by 31.1–31.9% as compared to their respective well-watered counterparts on day 10 of the induced drought. Thus, almost similar SWC among the stressed accessions indicates that different accessions have differential strategy to prevent water loss (inferred from RWC) together with growth cessation (Fig. 3b) in the early phase of the drought, and 10 days of drought is non-lethal in the investigated *A. thaliana* accessions under given experimental conditions.

### Strategy to avoid prior dark-adaptation in screening experiments: a comparison of ChlF emission of two contrasting accessions

**Fig. 4:**
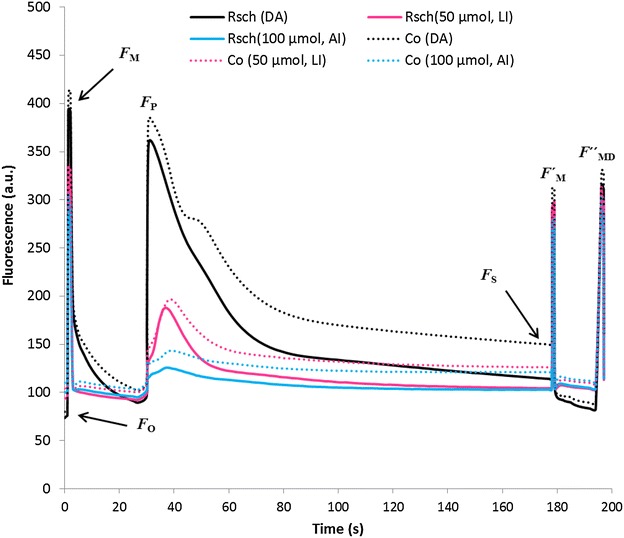
Chlorophyll *a* fluorescence (ChlF) transients of two contrasting *A. thaliana* accessions, Rsch (*solid line*) and Co (*dotted line*) measured after ~20 min of dark-adaptation (DA, *black lines*), and after 2 h of acclimation to *half of the adaptive irradiance* (LI, 50 µmol photons m^−2^ s^−1^, magenta lines) or *adaptive growth*-*irradiance* (AI, 100 µmol photons m^−2^ s^−1^, *blue lines*). The basic parameters: minimal fluorescence (*F*_O_), maximum fluorescence (*F*_M_), peak under prevailing actinic irradiance (*F*_P_), steady state fluorescence under actinic light (*F*_S_), maximum fluorescence at steady state fluorescence ($F_{\text{M}}^{\prime }$), and maximum fluorescence during relaxation after switching off of actinic light ($F_{\text{M}}^{\prime \prime }$), are indicated in the measured ChlF of dark-adapted plants. Each curve is an average of four data sets measured from independent plants and averaged over the whole rosette area

In order to examine the possibility of avoiding prior dark-adaptation in the protocol of ChlF measurements, we measured ChlF transients from 7-week old well-watered plant rosettes of two contrasting *A. thaliana* accessions, Rsch (Fig. 4, solid line) and Co (Fig. 4, dotted line), after 20 min of dark-adaptation, and immediately after ~2 h of acclimation to ~100 µmol photons m^−2^ s^−1^ (*adaptive growth*-*irradiance*) or ~50 µmol photons m^−2^ s^−1^ (*half of the adaptive growth*-*irradiance*). The associated ChlF parameters for both the accessions are shown in Table 1. We did not observe significant difference in the ChlF parameters, dark-adapted *minimum fluorescence* (*F*_O_), *steady state fluorescence* *F*_S_(L_1_ − L_2_) under *adaptive growth*-*irradiance* or under *low ambient*-*irradiance,* and the intensity of peak *F*_P_, between these two accessions; however, it is obvious from Fig. 4 that qualitative differences between the two accessions lie in the slow phase of ChlF transients, beyond peak *P*(*F*_P_), and it continued until *F*_S_.

**Table 1:** Basic chlorophyll *a* fluorescence (ChlF) parameters of two contrasting *A. thaliana* accessions, Rsch and Co, measured after 20 min of dark adaptation (DA), and immediately after 2 h of acclimation to low *growth-irradiance* (L_50_, ~50 μmol photons m^−2^ s^−1^ “*half of the adaptive* *growth*-*irradiance*”) and adaptive growth irradiance (L_100_, ~100 μmol photons m^−2^ s^−1^)

ChlF parameters	DA		L_50_ (50 μmol photons m^−2^ s^−1^)		L_100_ (100 μmol photons m^−2^ s^−1^)	
	Rsch	Co	Rsch	Co	Rsch	Co
*F*_O_	75 ± 4	81.5 ± 5	–	–	–	–
*F*_S_(L_1_)	–	–	94 ± 4	104 ± 10	100 ± 4	109 ± 10
*F*_P_	327 ± 20	355 ± 22	189 ± 22	197 ± 24	126 ± 2	144 ± 22
*F*_S_(L_2_)	115 ± 7	150 ± 8**	104 ± 6	126 ± 14	103 ± 4	121 ± 21
*F*_V_/*F*_M_	0.810 ± 0.003	0.803 ± 0.003	–	–	–	–
Φ_PSII_(L_1_)	–	–	0.714 ± 0.005	0.665 ± 0.008***	0.666 ± 0.004	0.610 ± 0.012**
Φ_PSII_(L_2_)	0.708 ± 0.005	0.634 ± 0.004***	0.683 ± 0.003	0.596 ± 0.010***	0.657 ± 0.006	0.570 ± 0.022**
NPQ	0.269 ± 0.001	0.244 ± 0.014**	0.251 ± 0.012	0.304 ± 0.071	0.295 ± 0.031	0.351 ± 0.082
NPQ (L)	–	–	0.105 ± 0.014	0.080 ± 0.012	0.075 ± 0.020	0.049 ± 0.013*
*R*_FD_	0.54 ± 0.01	0.74 ± 0.01***	1.33 ± 0.18	1.92 ± 0.28*	4.54 ± 0.48	6.03 ± 1.05*

The parameters shown are mean ± SE (n = 4/5)

Asterisks in the columns of Co for DA, for L_50_, and for L_100_ denote statistical significance of ChlF data for Rsch versus Co, respectively, measured after DA, and immediately after acclimation to 50 μmol photons m^−2^ s^−1^ (*low*) and 100 μmol photons m^−2^ s^−1^ (*adaptive*) light (* p < 0.05; ** p < 0.01; *** p < 0.001, unpaired t test)

Following acclimation to ~2 h of *adaptive growth*-*irradiance*, the *F*_P_ of Rsch and Co declined to 61 and 59%, as compared to that measured in dark-adapted plants (Table 1). However, when they were acclimated to *half of the adaptive growth irradiance*, the decline in *F*_P_ was much lower, 42 and 45%, for Rsch and Co, respectively (Table 1). Decline of *steady state fluorescence* *F*_S_(L_2_) was observed in both the accessions following acclimation to *low or adaptive* growth-irradiance as compared to that of dark-adapted samples (Table 1). For dark-adapted ChlF transients the difference in the mean value of *F*_S_(L_2_) for Rsch versus Co was 30%; this difference was reduced by 21 or 17% when measured immediately following 2 h of acclimation to *low* or *adaptive* growth-irradiance, respectively.

The *F*_V_/*F*_M_ had almost similar value (~0.803–0.810) in both Rsch and Co. However, the parameter Φ_PSII_(L_1_) measured for light adapted plants was significantly different (7–9%, p < 0.01) in Rsch versus Co, following ~2 h of acclimation of *low* and *adaptive* irradiance respectively (Table 1).

Comparison of unpaired t test for Φ_PSII_(L_2_) revealed that for dark adapted Rsch versus Co had an ~10% significant difference that further diverged by ~13% after ~2 h of acclimation to both *low* and *adaptive* irradiance (Table 1).

The difference in the mean value of NPQ between Rsch and Co for dark-adapted plants was only ~9%, but it was statistically significant (*p* < 0.01). This difference in NPQ for Rsch versus Co was increased to 19–21%, when measured following acclimation to *low* and *adaptive growth*-irradiance; however, it was non-significant (*p* > 0.05) because of high variability in its value post illumination (Table 1).

The maximum light acclimation induced changes was quite large in the ChlF parameter, fluorescence decrease ratio, *R*_FD_ (Table 1). For Rsch, *R*_FD_ changed to 146 and 741%, while for Co it changed to 159 and 715%, respectively, on acclimation to *low* and *adaptive growth*-*irradiance*, as compared to that of their dark-adapted values. However, differences in the *R*_FD_ for Rsch versus Co was 37, 44 and 33%, respectively, for dark-adapted, for adapted to *half of the growth-irradiance and* for *adaptive* growth-irradiance (Table 1).

### ChlF emission for screening leaf-water deficit

Comparison of ChlF transients and associated parameters of two contrasting accessions, Rsch and Co, convincingly demonstrates that the ChlF transients measured in the presence of *low ambient irradiance* (i.e., *half of the adaptive growth*-*irradiance*, 50 µmol photons m^−2^ s^−1^) not only differentiate two accessions but also preserve several important features comparable to that measured from dark-adapted plants; therefore, similar protocols might be used to screen plant traits without any dark-adaptation in greenhouses or under field situations. In order to validate the potential of this protocol, we measured ChlF transients of six well-watered and drought-stressed *A. thaliana* accessions using transect ChlF imaging system, in the presence of *low ambient irradiance*, with an aim to find out the features of ChlF emission that can be correlated with drought induced changes in leaf-water deficit measured by RWC.

**Fig. 5:**
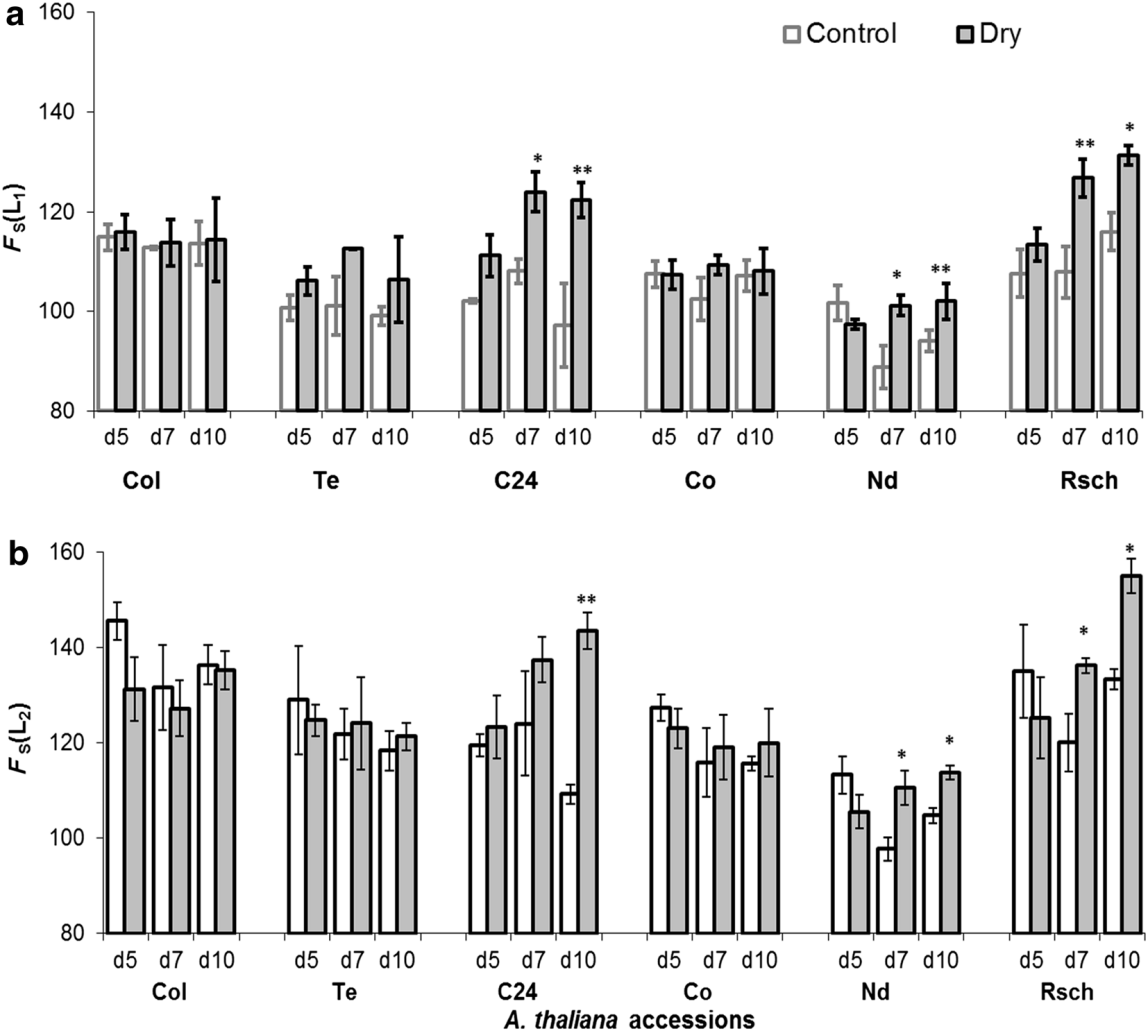
Steady state chlorophyll *a* fluorescence measured in the presence of **a** low ambient irradiance ~50 µmol photons m^−2^ s^−1^ [*F*_S_(L_1_)] and **b** actinic irradiance ~150 µmol photons m^−2^ s^−1^ [*F*_S_(L_2_)]. The values shown in the figure are mean of three independent plants ± SE (n = 3). *d5* (day 5), *d7* (day 7), and *d10* (day 10). Asterisks denote significant differences of drought-stressed from well-watered plants (*p < 0.05; **p < 0.01; unpaired t test)

Figure 5 shows the changes in *steady state fluorescence*, *F*_S_, for well-watered and drought-stressed accessions measured after acclimation to *low ambient irradiance* of 50 µmol photons m^−2^ s^−1^ [*F*_S_(L_1_), Fig. 5a], and under *actinic*-*irradiance* ~150 µmol photons m^−2^ s^−1^ [*F*_S_(L_2_), Fig. 5b]. Both [*F*_S_(L_1_), *F*_S_(L_2_)], parameters rose significantly in drought-stressed accessions C24, Nd and Rsch on day 10 of the induced drought. However, *F*_S_(L_1_) seems more sensitive for measuring drought responses as it showed significant rise also on day 7 in all three accessions, while *F*_S_(L_2_) on day 7 was not different for C24.

**Fig. 6:**
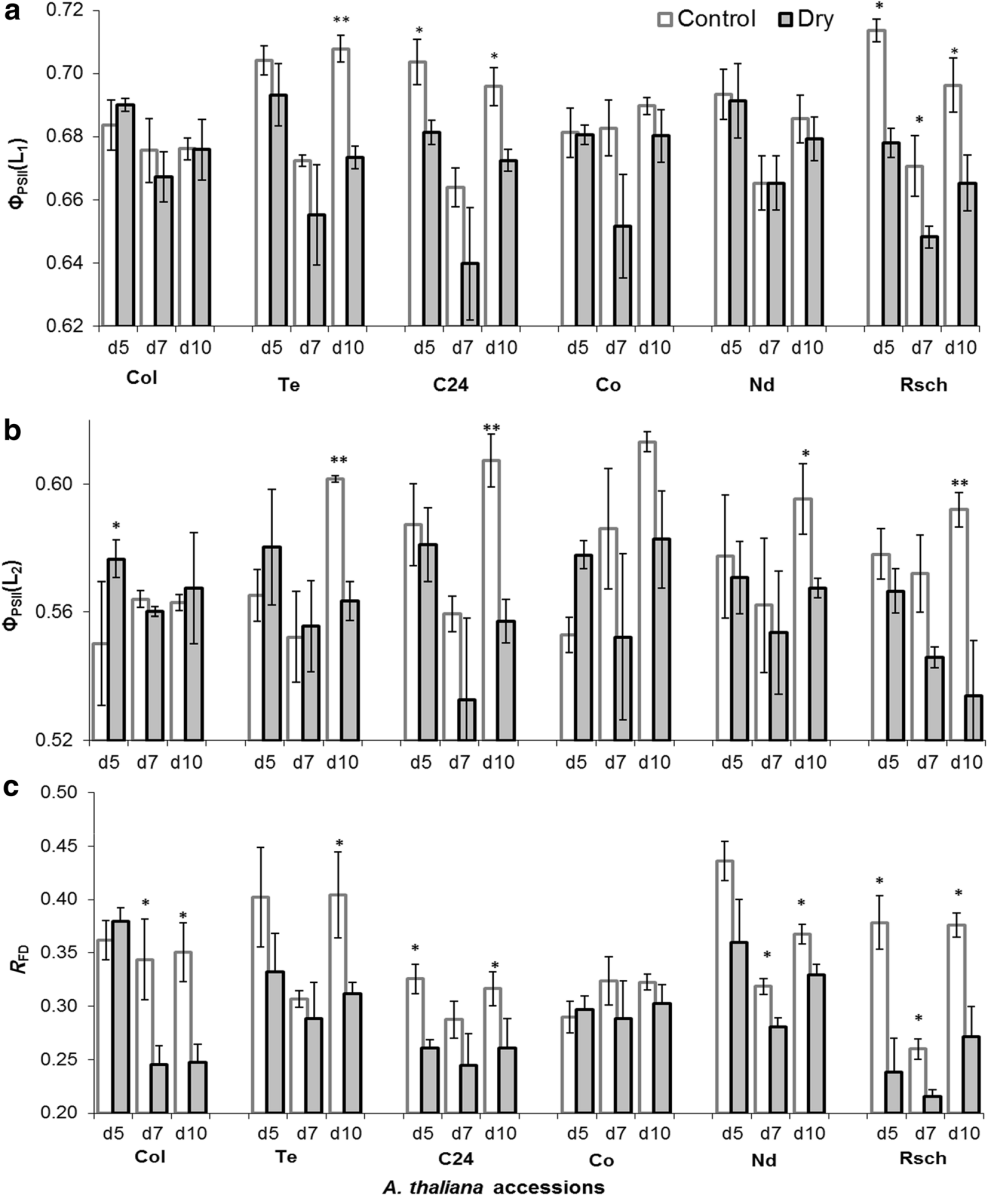
Chlorophyll *a* fluorescence parameters: effective quantum efficiency of PSII in **a** *low ambient irradiance* [Φ_PSII_(L_1_)], in **b** under *actinic irradiance* ~150 µmol photons m^−2^ s^−1^ [Φ_PSII_(L_2_)], and **c** *fluorescence decrease ratio* (*R*_FD_). The values shown here are mean of results from three independent plants with standard errors. *d5* (day 5), *d7* (day 7), and *d10* (day 10). *Asterisks* denote significant differences between drought stressed and well-watered plants (*p < 0.05; **p < 0.01; unpaired t test)

The value of *F*_V_/*F*_M_ ranged between 0.80 and 0.83 in all the accessions and there was insignificant difference for well-watered versus drought-stressed plants during 10 days of induced-drought (data not shown). However, Φ_PSII_(L_1_) significantly decreased in drought-stressed Rsch as compared to control plants on day 5 of the induced drought (Fig. 6a). On day 10 of the induced drought, when RWC was significantly decreased in all drought-stressed accessions, the ChlF parameters Φ_PSII_(L_1_) and Φ_PSII_(L_2_) were significantly reduced in three (Rsch, Te, and C24; Fig. 6a) and in four (Rsch, Te, C24, and Nd; Fig. 6b) accessions, respectively, as compared to their well-watered counterparts.

The ChlF parameter, *R*_FD_ (Fig. 6c), significantly decreased in five accessions (Rsch, Nd, Col, Te and C24) on day 10 of induced stress. Interestingly, *R*_FD_ differed significantly for drought-stressed versus well-watered Rsch (having high RWC difference) plants on day 5, day 7 and day 10 of the induced drought, while its values were almost the same for well-watered and drought-stressed Co (for which lowest difference in leaf RWC) plants until day 10 of the induced drought.

### Combinatorial imaging appears to find early features of leaf-water deficit

Based on the differences between the ChlF transients of well-watered versus drought-stressed plants having large differences in RWC among the group of accessions, training and performance testing of the various classifiers were executed. On day 5 of induced drought we observed that RWC in drought-stressed Rsch accession significantly (*p* < 0.05) differed from its well-watered counterparts (Fig. 2), and this adjustment was accompanied by changes in the parameters Φ_PSII_(L_1_) and *R*_FD_ (Fig. 6). During 10 days of the induced drought, maximum and minimum changes in RWC between well-watered and stressed plants, were obtained for Rsch (~16%) and Co (~7%) respectively (Fig. 2). Such changes in RWC revealed that among the investigated six accessions, leaves of Rsch and Co had the most and least features to preserve their leaf-water. Therefore, the training of six classifiers (QDC, LDC, NNC, k-NNC, SVC, and NeuC), were executed to obtain the discriminant features of leaf RWC from the time-series ChlF data of highly contrasting accessions Rsch and Co, measured on day 5 of induced drought. This was important because early diagnosis of features, such as leaf-water deficit or drought symptoms, is one of the main problems to be addressed in plant phenotyping. The evaluation of underlying parameters e.g., *performance*, *error rate* and *computational time* to run the algorithms unveiled that LDC is the best performing classifier (80% correct assignment of drought stressed features among the tested image data set) that completed data execution in a comparatively short time (~8.5 s, Table 2). Therefore, LDC was applied with sequential forward floating selection (SFFS) feature selection method for searching contrasting sets of ChlF images with inherently distinct features capable of distinguishing leaves having low and high RWC among the *A. thaliana* accessions. The algorithm of SFFS reduced the full data set of 216 images into three images identified as I_184_, I_112_ and I_39_, respectively, measured at ~182, 105 and 31 s, without compromising the classification performance (~79%). We obtained the linear combination of images: C = (−0.4136) × I_184_ + (+0.7937) × I_112_ + (−0.4163) × I_39_, to discriminate between drought-stressed plants in the *A. thaliana* accessions used in this research. The coefficients of the linear combination were calculated according to Pineda et al. [45].

**Table 2:** Performance and computational time required for the tested classifiers

Classifiers	Performance	Computational time (min)
QDC	0.66	6
LDC	0.80	8.5
NN	0.71	3613
k-NN	0.74	3396
SVC	0.75	15,420
NeurC	0.76	913

**Fig. 7:**
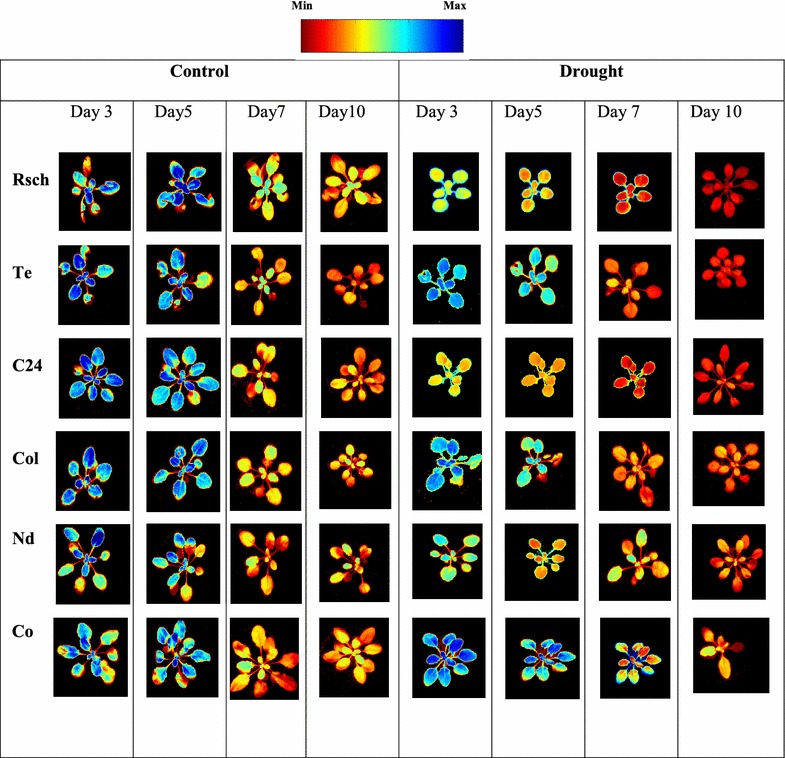
Combinatorial imaging showing the combination of three most contrasting images for six accessions of *A. thaliana*. These most contrasting images were chosen using combination of linear discriminant classifier (LDC) and sequential forward floating selection (SFFS). The training was done with Chl *a* fluorescence transient “captured” on day 5 of drought treatment. In this process, we obtained three coefficients for three images that gave the highest contrast between drought tolerant and sensitive accession. The images of all the accessions are shown to demonstrate that the classification works for them for day 5 as well as for day 3, day 7, and day 10 of the induced drought stress

In Fig. 7, we show representatives of the resulting combinatorial fluorescence images (*F*_CI_) for well-watered and stressed plants of six accessions on day 3, day 5, day 7, and day 10 of the induced drought. In Table 3, the corresponding mean pixel intensity, *F*_CI_, averaged over the rosettes area of three well-watered and three drought-stressed plants of all six accessions are presented. By comparing false colors in combinatorial images of control (left side panel of Fig. 7) and drought-stressed (right side panel of Fig. 7) plants or their corresponding averaged *F*_CI_ (Table 3) on day 5 of induced drought, it is obvious that fluorescence measurements have enabled us to visualize the features of leaf-water deficit in almost similar to what we had evaluated from RWC measurements (Fig. 2). On day 10 of induced drought when RWC significantly differentiated all drought-stressed accessions as compared to their respective well-watered (Fig. 2); unpaired t test confirmed that *F*_CI_ significantly differentiated four accessions not only on day 10 but also on day 7 of the induced drought as well (Table 3). On day 5 of the induced drought when RWC of accession Rsch was significantly different, we noticed significant difference of *F*_CI_ in accessions Te and Col not only on day 5 but also on day 3 of the induced drought for well-watered versus drought-stressed, suggesting a good use of this method for early sensing of drought induced changes in plant leaves.

**Table 3:** Mean of pixel intensity from derived combinatorial fluorescence images (*F*_CI_) for well-watered (control) and drought-stressed (dry) on day 3 (d3), day 5 (d5), day 7 (d7), and day 10 (d10) of the induced drought

*A. thaliana* accessions	Mean pixel intensity of derived combinatorial fluorescence images (*F*_CI_)			
	d3	d5	d7	d10
Rsch				
Control	160.6 ± 2.1	141.9 ± 3.5	139.2 ± 4.2	134.3 ± 4.2
Dry	118.6 ± 3.3***	120.1 ± 2.4**	105.6 ± 4.1**	61.1 ± 2.0***
Te				
Control	118.2 ± 2.1	121.8 ± 3.9	124.1 ± 4.5	109.3 ± 3.8
Dry	145.9 ± 4.4*	144.3 ± 7.9*	91.0 ± 7.0**	64.7 ± 7.4**
C24				
Control	142.2 ± 15.0	138.7 ± 1.4	135.4 ± 1.4	123.9 ± 3.5
Dry	113.4 ± 4.8	130.7 ± 7.4	104.0 ± 8.0**	68.9 ± 5.3***
Col				
Control	121.4 ± 2.6	129.1 ± 3.4	138.9 ± 1.9	129.8 ± 3.8
Dry	142.8 ± 1.8*	142.3 ± 4.02*	115.4 ± 5.3**	98.9 ± 5.4**
Nd				
Control	122.7 ± 5.2	132.6 ± 4.5	120.1 ± 7.1	112.9 ± 5.5
Dry	156.7 ± 0.8	147.1 ± 4.7	128.5 ± 10.2	97.7 ± 11.2
Co				
Control	133.6 ± 1.3	131.3 ± 1.9	124.6 ± 2.3	119.3 ± 7.8
Dry	124.6 ± 7.6	136.1 ± 12.8	140.5 ± 3.8*	129.5 ± 5.1

The data shown are mean over the whole rosettes ± SE (n = 3)

Asterisks (*) in the row of drought-stressed “dry” denote statistical significance of *F*_CI_ data with respect to their corresponding control plant samples (* *p* < 0.05; ** *p* < 0.01; *** *p* < 0.001, unpaired t test)

**Fig. 8:**
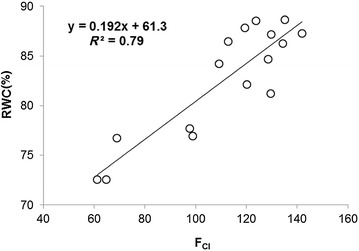
Correlation between leaf relative water content (RWC) and mean pixel intensity of combinatorial fluorescence images (*F*_CI_), for which a significant difference in RWC between well-watered and drought-stressed plants was obtained

For finding quantitative relationship between RWCs (Fig. 2) and *F*_CI_ (Fig. 7; Table 3), a scattered graph was plotted (Fig. 8). A regression line with coefficient of determination, *R* ^2^ = 0.79, was thus found. In Fig. 8, a mean of *F*_CI_ was chosen corresponding to all accessions during entire experiments for which statistically significant differences in RWC for well-watered versus drought-stressed were obtained.

## Discussion

### Drought responses in natural accessions of *A. thaliana*

Since drought induces many highly complex responses that include molecular, biochemical, physiological, and morphological changes, which are dependent on the species and their developmental stage, selection of natural accessions of the model plant *A. thaliana* that grow over large geographical regions and are exposed to diverse environmental conditions, was for us, an interesting choice for testing ChlF based methods in view of the early sensing of drought responses in this species. In general, it is assumed that methods tested on model species are valuable and applicable to other plant species as well. The differential response of *A. thaliana* accessions to the drought as represented in RWC changes (Fig. 2) is not surprising as the response may involve coordinated changes in RNA transcription, developmental timing, growth allocation, sugar metabolism, cell wall composition, cytosolic chemistry, and photosynthetic activity. However, we note that the degree of changes in drought avoidance and tolerance mechanisms may be quite different, primarily because of genetic variations [63]. These variations, however, make it possible to trace corresponding changes in ChlF transient or its parameters for revealing underlying mechanisms.

### Light adaptive advantage in plants can be used for throughput amplification of ChlF emission measurements

Our study has convincingly revealed the possibility to exploit light adaptive advantage of ChlF emission measurements that had allowed quick throughput monitoring of many plants. Light is highly dynamic in nature, varying several fold in a single day and photosynthetic organisms have developed a number of mechanisms to adjust their photosynthesis, physiology, and ways to retain equilibrium while intercepting light energy, funneling of excitation energy to the photosynthetic reaction centers, formation of NADPH and ATP within the thylakoid membranes, and their utilization for CO_2_ fixation, as well as for the biosynthesis of primary and secondary metabolites [64, 65]. Lowering *adaptive growth*-*irradiance* to half of its value slows down the rate of regulation of photosynthetic reactions and associated enzymatic activities in the investigated accessions, and the presence of significant number of open reaction centers under *low ambient irradiance* possibly responsible for retaining of typical shape of ChlF transients (Fig. 4). Following acclimation to *low ambient irradiance* the divergence between well-watered plants of two contrasting accessions, Rsch and Co, was not statistically significant in many of the ChlF parameters (e.g., *F*_P_, *F*_S_, NPQ; see Table 1); however, several of their features (e.g., dynamic behavior of ChlF transients, Φ_PSII_, *R*_FD_; see Fig. 4; Table 1) were preserved and comparable to those measured after dark-adaptation. This demonstrates that under *low ambient irradiance*, slight differences in inherent genetic characteristics, or may be photosynthetic efficiency, can be probed by ChlF transients and associated parameters. Thus, we may use this method to detect the occurrence of drought induced systematic changes in plants.

Plants acclimated to short term *low irradiance* (here it is half of the *adaptive growth*-*irradiance*) yielded a well-defined *steady state fluorescence* [*F*_S_(L_1_)] and *effective quantum efficiency of PSII* [Φ_PSII_(L_1_)], and these parameters provided better discrimination capacity as compared to those measured under actinic light, e.g., *F*_S_(L_2_) and Φ_PSII_(L_2_) (Figs. 5, 6). The Φ_PSII_, measures indirectly photosynthetic performance under the prevailing light [66]; however, Φ_PSII_(L_1_) depends on *F*_S_(L_1_) with partially reduced Q_A_ and slower rate of redox reactions. The measured *F*_P_, in the ChlF transients, under *low ambient irradiance*, enabled us to determine another important parameter, *fluorescence decrease ratio* (*R*_FD_) [67, 68]. Therefore, we propose to consider routine screening of ChlF emission to access adaptive significance of plant photosynthesis since this enables us to screen large number of plants and that may ultimately allow better understanding of photosynthetic performance in many genotypic traits.

### ChlF for sensing early features of drought induced changes in leaf-water deficit

Despite complexity in signaling pathways at metabolomic, biochemical and physiological aspects, while understanding drought tolerance and avoidance mechanisms, there is a consensus that onset of drought reduces transpirational water loss via stomatal closure, which further reduces intracellular CO_2_ concentration, water potential, and RWC, and altogether they impair photosynthetic activity. Severe drought exposure leads to C/N imbalance and may disrupt organelles, e.g., chloroplast and plasma membranes, and cause senescence or even death [69]. Thus, developing robust and non-invasive methods for precise measurement of highly complex drought tolerance and/or avoidance in plants is highly challenging, also because continuously growing plants may aggravate systematic (caused by growth) and/or dynamic (caused by diurnal variations of photosynthetic activity) errors in the measured data while being under stress. The systematic and dynamic errors might cause random fluctuations of several empirical ChlF parameters of even well-watered (controls) accessions measured at different days of induced drought (Figs. 5, 6). Comparing RWC, a direct measure of leaf-water deficit, to the non-invasive light adapted ChlF parameters, and combining them to combinatorial images (trained on images pixels of accessions having differential RWC response during drought stress) to investigate the features of their correspondence, was the main objective of this research. Earlier investigations showed that dark-adapted ChlF parameter, *F*_V_/*F*_M_, was very stable during mild to moderate drought and when it changed leaves were already lethally damaged [22, 23, 55]. High stability of *F*_V_/*F*_M_ during prolonged drought stress may be because it is calculated from the parameters, *F*_O_ and *S*_M_, measured under extreme conditions, i.e., after a long dark-adaptation when primary electron acceptor Q_A_ is fully oxidized and reduced respectively [27, 51]. Light acclimation induces various mechanisms, e.g., non-photochemical quenching and photoinhibition [54], affecting dynamics of molecular reactions responsible for changes in steady state fluorescence (*F*_S_) and maximum fluorescence under prevailing light conditions $F_{\text{M}}^{\prime } ),$ and all this caused large variations in parameter Φ_PSII_. Therefore, the molecular mechanisms responsible for short term light acclimation induced change in the steady state fluorescence [*F*_S_(L_1_) or *F*_S_(L_2_)] could be different for diverse accessions during water deficit. However, it was interesting to note that high divergence of RWC for stressed accessions Rsch, Te, and C24 as compared to that of their well-watered plants (Fig. 2) resembled the almost similar degree of decline in Φ_PSII_(L_1_) and Φ_PSII_(L_2_) (Fig. 6). This indicates that stomatal closure and further inhibition of CO_2_ supply to the leaf chloroplast might be the reason for causing high divergence of Φ_PSII_(L_1_) as well as of Φ_PSII_(L_2_) for well-watered versus drought-stressed accessions of Rsch, Te, and C24. Those accessions which showed insignificant change in Φ_PSII_(L_1_) and Φ_PSII_(L_2_), also had less divergence in RWC, suggesting incident actinic light energy was almost optimally utilized by CO_2_ fixation and photorespiration processes [70, 71]. Omasa and Takayama [41] reported that Φ_PSII_ did not change regardless of stomatal closure if the actinic light was fully utilized in photosynthetic pathway. However, they observed that Φ_PSII_ declined in the presence of high actinic irradiance and reported that the non-photochemical mechanism can be rapidly activated if the absorbed light energy exceeds the energy consumption by CO_2_ fixation and photorespiration.

### Pattern-recognition based combinatorial imaging for early diagnosis of leaf-water deficit

Combinatorial imaging is based on pattern-recognition algorithms, and it analyses pixel data sets of the time-series ChlF image sequence for information extraction; it has already been demonstrated to be superior over classical ChlF parameters in visualizing early detection of biotic stress [42–44], in discriminating plant species of the same family at very young stage of their growth [47], and in investigating features for cold tolerance at non-lethal temperatures [48, 49]. As early detection of leaf-water deficit or finding symptoms of drought was a key question in screening experiment, the training of the different classifiers and feature selection methods on accessions Rsch and Co, having contrasting RWC on day 10 of induced drought, was necessary. We found that *F*_CI_ (Fig. 7; Table 3) obtained by this method yielded a very good correspondence between well-watered and drought-stressed accessions not only on day 5 (when RWC was significantly different for Rsch well-watered versus drought stressed, Fig. 2) but it continued to discriminate accessions on the basis of their RWC until day 10 of the induced drought. Usually ChlF images are highly heterogeneous across the leaf [40–43]; however, *F*_CI_ gave statistically significant difference between the well-watered and drought-stressed plants for four accessions not only on day 10 but also on day 7 of the induced drought; further, a reasonably high correlation coefficient factor *R* ^2^ = 0.79 (Fig. 8) between RWC and *F*_CI_ indicates that *F*_CI_ composed of inherent features of drought and may be used as proxy of early drought affects in plants. The derived combinatorial images (Fig. 7) or its corresponding *F*_CI_ values (Table 3) of well-watered accessions measured on day 7 and on day 10 are very different than that of the images/values measured on day 3 and day 5, and their pixel intensity seems adjacent to that obtained for stressed accessions. We note that training of the data sets was executed on stressed Rsch and Co, having high difference on RWC already on day 5 of the induced drought; therefore, high error in the derived image of continuously growing control plants is obvious as its data were not included in the training data sets.

## Conclusions

As far as we know, this is the first time anyone has established that the natural adaptive adjustment of plants to growth-irradiance can be exploited to measure typical ChlF transients in the presence of *low ambient irradiance* without any prior dark-adaptation, and the measured ChlF transients preserved the information comparable to those obtained in dark-adapted plants. The new protocol not only improves the throughput of the measurements, but also adds physiologically relevant parameters: *steady state fluorescence* (*F*_S_), *effective quantum efficiency of PSII* (Φ_PSII_), and *fluorescence decrease ratio* (*R*_FD_), and, thus, these ChlF parameters could be directly exploited in breeding programs. The combinatorial imaging after being trained on contrasting accessions, having large difference in RWC of leaves, could identify drought induced symptoms at least in the early stage of drought progression in *A. thaliana* accessions. This opens up the possibility to use the adaptive significance of plants for measuring full ChlF transients at fairly high-throughput scale in the greenhouses or in the field conditions, and after integration with combinatorial image analysis tools this method can be applied to sense initiation of drought stress. Such a system will be very useful for phenotypic screening of drought or other biotic/abiotic stresses, e.g., in recombinant inbred line populations or other plant sets consisting of individual plants representing different genotypes.

## Abbreviations

ChlF: chlorophyll *a* fluorescence
Φ_PSII_: effective quantum efficiency of PSII [Φ_PSII_ = $(F_{\text{M}}^{\prime } - {F}_{\text{S}} )$/$F_{\text{M}}^{\prime }$]
$F_{\text{M}}^{\prime }$: maximum fluorescence in light adapted state
*F*_S_: steady state fluorescence
*R*_FD_: fluorescence decrease ratio [*R*_FD_ = *F*_D_/*F*_S_(L_2_), where, *F*_D_ = *F*_P_ − *F*_S_(L_2_)]
*F*_P_: the maxima in ChlF transients under actinic irradiance
*F*_S_(L): steady state fluorescence under *irradiance* L (L_1_/L_2_ = 50/150 µmol photons m^−2^ s^−1^)
*F*_V_/*F*_M_: maximum quantum yield of PSII photochemistry
RWC: relative water content
PS: photosystem
Q_A_: primary quinone acceptor
*F*_O_: minimal fluorescence emission of a dark adapted plant with Q_A_ oxidized and non-photochemical quenching inactive
*F*_M_: maximal fluorescence emission intensity of a dark adapted plant exposed to a short pulse of a strong light leading to a transient reduction of Q_A_ without induction of non-photochemical quenching
NPQ: non-photochemical quenching [NPQ = (*F*_M_ − $F_{\text{M}}^{\prime }$)/$F_{\text{M}}^{\prime }$]
*F*_V_: variable fluorescence (*F*_V_ = *F*_M_ − *F*_O_)
LED: light-emitting-diode
SWC: soil water content
MF: measuring flash
AL: actinic light
SF: saturating flash
Φ_PSII_(L): effective quantum efficiency of PSII at irradiance L
LDC: linear discriminant classifier
QDC: quadratic discriminant classifier
NNC: nearest neighbor classifier
k-NNC: *k*-nearest neighbors classifier
NMC: nearest mean classifier
SVC: support vector classifier
NeurC: neural network classifier
SFFS: sequential forward floating selection
